# The Audiovisual Tau Effect in Infancy

**DOI:** 10.1371/journal.pone.0009503

**Published:** 2010-03-03

**Authors:** Takahiro Kawabe, Nobu Shirai, Yuji Wada, Kayo Miura, So Kanazawa, Masami K. Yamaguchi

**Affiliations:** 1 Institute for Advanced Study, Kyushu University, Fukuoka, Fukuoka, Japan; 2 Department of Psychology, Niigata University, Niigata, Niigata, Japan; 3 Sensory and Cognitive Food Science Laboratory, National Food Research Institute, Tsukuba, Ibaraki, Japan; 4 Faculty of Human-Environment Studies, Kyushu University, Fukuoka, Fukuoka, Japan; 5 Department of Psychology, Japan Women's University, Kawasaki, Kanagawa, Japan; 6 Department of Psychology, Chuo University, Hachioji, Tokyo, Japan; 7 Precursor Research for Embryonic Science and Technology, Japan Science and Technology Agency, Kawaguchi, Saitama, Japan; Macquarie University, Australia

## Abstract

**Background:**

Perceived spatial intervals between successive flashes can be distorted by varying the temporal intervals between them (the “tau effect”). A previous study showed that a tau effect for visual flashes could be induced when they were accompanied by auditory beeps with varied temporal intervals (an audiovisual tau effect).

**Methodology/Principal Findings:**

We conducted two experiments to investigate whether the audiovisual tau effect occurs in infancy. Forty-eight infants aged 5–8 months took part in this study. In Experiment 1, infants were familiarized with audiovisual stimuli consisting of three pairs of two flashes and three beeps. The onsets of the first and third pairs of flashes were respectively matched to those of the first and third beeps. The onset of the second pair of flashes was separated from that of the second beep by 150 ms. Following the familiarization phase, infants were exposed to a test stimulus composed of two vertical arrays of three static flashes with different spatial intervals. We hypothesized that if the audiovisual tau effect occurred in infancy then infants would preferentially look at the flash array with spatial intervals that would be expected to be different from the perceived spatial intervals between flashes they were exposed to in the familiarization phase. The results of Experiment 1 supported this hypothesis. In Experiment 2, the first and third beeps were removed from the familiarization stimuli, resulting in the disappearance of the audiovisual tau effect. This indicates that the modulation of temporal intervals among flashes by beeps was essential for the audiovisual tau effect to occur (Experiment 2).

**Conclusions/Significance:**

These results suggest that the cross-modal processing that underlies the audiovisual tau effect occurs even in early infancy. In particular, the results indicate that audiovisual modulation of temporal intervals emerges by 5–8 months of age.

## Introduction

Human perception is fundamentally multimodal. Information in each input modality from different sensory receptors is integrated in the brain, leading to an internal construction of perceptual events. In the present study, we addressed the issue of whether the perceptual system of 5- to 8-month-old infants is able to construct audiovisual events by arbitrarily integrating auditory and visual information across space and time.

Previous studies have investigated several aspects of audiovisual integration in infants. For example, it has been found that infants are able to detect temporal concurrency of audiovisual information [1], [2], [3], and to match intensity across information [4]. These types of audiovisual interaction have been classified into amodal crossmodal interactions (i.e. amodal interactions for simplicity) because the interactions are relevant to the detection of amodally invariant relations between sensory signals [3], [5] in a convergent physical dimension (such as time or intensity). However, it has been suggested that these data may simply reflect the detection of amodal equivalence of signals perceived through several input modalities. For example, it has been shown in two studies [6], [7] that infants are able to detect equivalence in temporal congruency or intensity. It has been proposed that such early cross-modal coherency is likely to reflect undifferentiated sensory pathways in young infants [8], [9].

In addition to amodal interactions, crossmodal interactions that are relevant to nonlinear, orthogonal or arbitrary relations between sensory signals have also been examined [3], [5], [10]. These interactions have been classified into arbitrary crossmodal interactions [5], [11], [12] (i.e. arbitrary interactions for simplicity), referring to the phenomenon whereby a cross-modal manipulation in one dimension (temporal or spatial) alters the perceptual experience of multimodal stimuli in the other dimension (spatial or temporal). One example of arbitrary interactions is the audiovisual stream/bounce illusion [13]–[15] whereby the temporal congruence of audiovisual signals alters the perception of the spatial dimension of motion (i.e. motion trajectories).

Recent studies have provided evidence of arbitrary interaction in infancy. For instance, it has been demonstrated that infants aged over 6 months are able to perceive the audiovisual stream/bounce illusion [16]. Another study found that 10-month-old infants could use the consequences of arbitrary interaction of audiovisual information to orient their head and gaze towards an audiovisual target, whereas infants under 8 months of age could not [12]. Moreover, it has been demonstrated that a salient auditory pulse enhances infants' detection of a unique visual object (a subjective contour) in a rapid sequence of visual displays with ‘Pac-man’-shaped figures [17], as has also been observed in adults [18]. These findings demonstrate that even in infants older than 6 months audiovisual information is processed in complex ways, similar to that in adults.

In the present study we focused on a phenomenon of arbitrary interaction of multimodal information, the ‘audiovisual tau effect’ [19]. The tau effect refers to the phenomenon whereby perceived spatial intervals of successive flashes are affected by the temporal intervals between them. For instance, a spatial interval of two successive visual flashes is typically perceived as longer or shorter when the temporal interval between them is longer or shorter than their actual duration. The tau effect has been observed in visual [20], auditory [21], and tactile perception [22], [23]. A recent study by our research group investigated whether the tau effect is based on audiovisual interaction [19]. Three-stroke apparent motion composed of three successive visual flashes (F1, F2, and F3, in order) was used as a stimulus, accompanied by a sequence of three transient beeps (B1, B2, and B3, in order). The onsets of the first and last (third) sound were completely synchronized with those of the first and last visual object, while the onset of the second sound was temporally displaced from that of second visual object. It was found that when the first temporal interval between B1 and B2 was shorter than the second temporal interval between B2 and B3, the first spatial interval between F1 and F2 was perceived to be shorter than the second spatial interval between F2 and F3. On the other hand, when the first temporal interval between B1 and B2 was longer than the second temporal interval between B2 and B3, the first spatial interval between F1 and F2 was perceived to be longer than the second spatial interval between F2 and F3. Importantly, this effect disappeared when B1 and B3 were withdrawn from the stimulus. These findings suggest that the audiovisual tau effect arises from the modulation of temporal intervals between flashes by those between concurrent beeps, not simply from the temporal capture occurring between F2 and B2. As such, the audio-visual tau effect appears to be a typical example of arbitrary interaction in which auditory “temporal” interval alters visual “spatial” interval.

In the present study, we conducted two experiments to examine whether the audiovisual tau effect occurs in infancy. Two things would be assessed by testing the audiovisual tau effects in infancy. First, we assessed whether audiovisual modulation of temporal interval existed in infancy: The audiovisual tau effect entails it [19]. Second, as described above, we assessed whether arbitrary audiovisual interactions across space and time perception existed in infancy. In Experiment 1, we aimed to determine whether perceived spatial interval between successive flashes was modulated by temporal intervals between accompanying beeps. In Experiment 2, we confirmed that the temporal separation of the onsets between the second beep and the second flash was not responsible for the audiovisual tau effect in infancy. As a result, consistent with findings obtained in adults [19], [24], audiovisual interaction in temporal dimension altered spatial appearances of visual signals in infants.

## Materials and Methods

### Ethics Statement

Ethical approval for this study was obtained from the ethical committee at the National Food Research Institute. Moreover, the experiments were conducted according to the principles laid down in the Helsinki declaration. Written informed consent was obtained from each infant's caregiver prior to participation in the experiment.

### Experiment 1

#### Participants

A total of 24 5- to 8-month-old infants (mean = 194.5 days, standard deviation [SD] = 33.3) took part in Experiment 1. The infants were selected from the participants-database of the infant laboratory, Chuo University (Tokyo, Japan). Local newspaper advertisements were used to recruit infants and their families. Only infants of families who voluntary contacted the infant laboratory were enrolled to the database. All infants were healthy full-terms that weighed >2500 g at birth. Two additional infants took part in this experiment, but their data were excluded from the analysis because experimental sessions were discontinued due to crying and extreme side bias (100% looking to the right side of display) in the test phase described below.

#### Apparatus

All visual stimuli were displayed on a 21-inch color CRT monitor (Nanao Flexcan T966) with a resolution of 1024×768 pixels, an 8-bit color mode, and a refresh rate of 60 Hz. Presentation of stimuli was controlled by an IBM-compatible PC (Dospara Galleria Series). Two loudspeakers were positioned on either side of the monitor for the presentation of auditory stimuli. A CCD camera, which was attached just below the CRT and connected to a video deck and a TV monitor, was used to record the behavior of each participant, and to observe them on-line.

#### Stimuli

We used a familiarity-novelty preferential looking technique; a popular experimental technique to investigate infants' ability to discriminate between different visual stimuli (see the review parts of [25], [26]). This technique relies on the fact that infants tend to show visual preference for novel stimuli [27].

**Figure 1:**
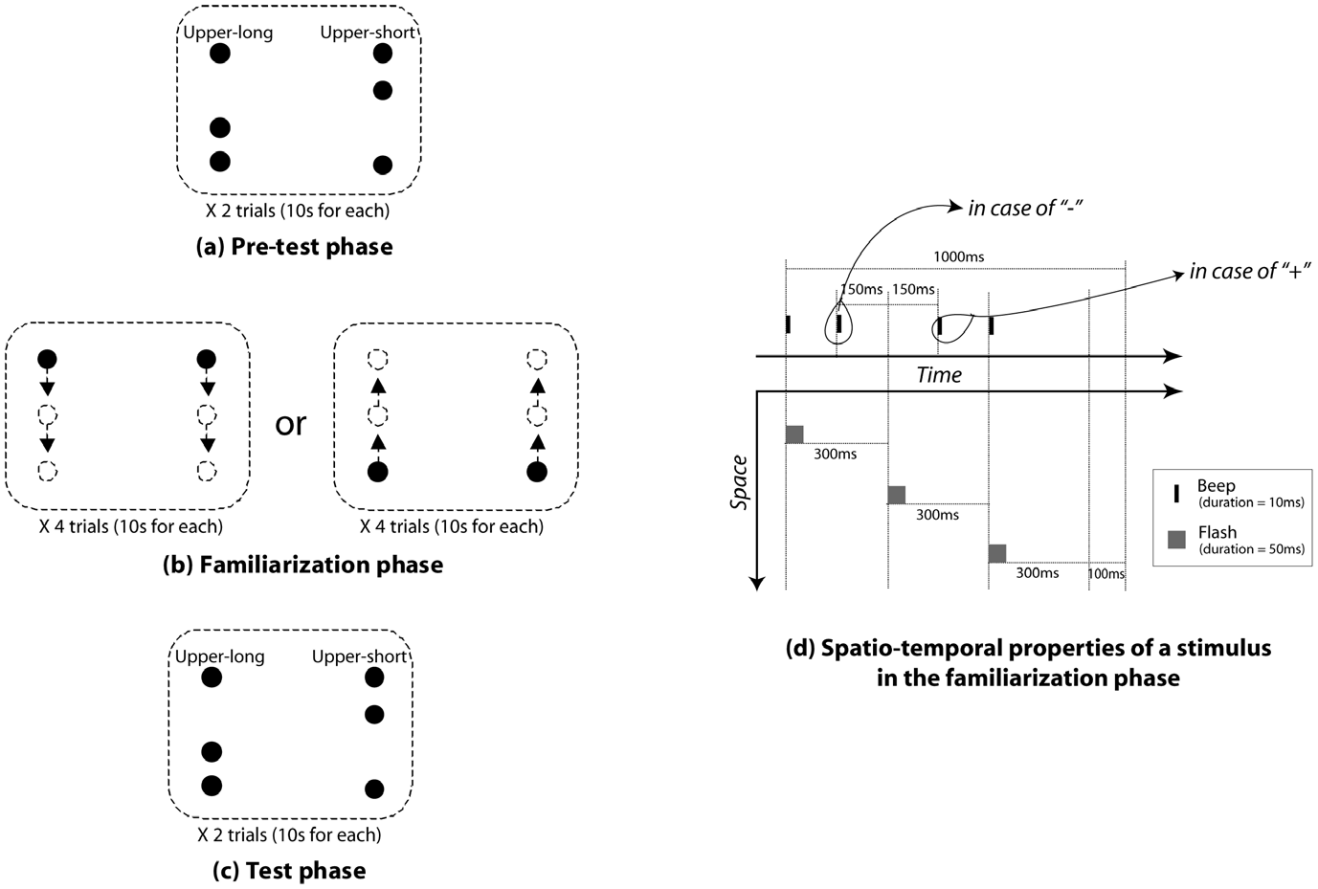
Schematic illustrations of stimuli (a) in the familiarization phase and (b) in the test and (c) pre-test phase. (d) A chart representing spatiotemporal properties of stimuli presented in the familiarization phase.

In the familiarization phase, each stimulus consisted of six successive flashes and three auditory beeps (Fig. 1b). Each flash was disk-shaped, and its duration, diameter, and luminance were 50 ms, 2.3 deg, and 3.3 cd/m^2^, respectively. The flash was presented against a white presentation field with a size of 57.3×43.0 deg, and luminance of 102 cd/m^2^. Each of the flashes in the first pair was presented at the left and right side of the display with a horizontal eccentricity of 20.2 deg. The first pair of flashes was followed by the second and third pairs with an inter-stimulus interval (ISI) of 250 ms and a spatial interval of 13.4 deg. Flashes within each pair were horizontally separated by 40.3 deg. When the first pair was presented 13.4 deg above (or below) the vertical center of display, the second and third pairs were sequentially presented 0 deg and 13.4 deg below (or above) the vertical center of display (i.e. downward and upward conditions, respectively). These two conditions were counterbalanced across infants. Each beep was a pure-tone burst. Its duration and frequency were 10 ms and 440 Hz, respectively. Beeps were accompanied with flashes. The onsets of the first and third beeps were completely synchronized with those of the first and third pairs of flashes, while the onset of second beep preceded (or followed) the onset of the second pair of flashes by 150 ms (Fig. 1d). At the initiation of each trial, a white blank display with no sound was presented for 100 ms, immediately followed by the sequence of flashes and beeps. Sets consisting of the blank display plus one sequence of flashes-beeps were displayed cyclically. Each cycle lasted for 1000 ms. Ten cycles were presented on each trial.

In the pre-test and test phases, two static vertical arrays of three flashes were presented (Figs. 1a and 1c). The size and luminance of each flash were identical to those in the familiarization phase. The spatial interval between flashes in each array was distorted in the following two ways: For one array, the spatial interval between the upper and middle flashes (15.1 deg) was longer than that between the middle and lower flashes (11.7 deg). We designated this array ‘upper-long’. For the other array, the interval between the upper and middle flashes (11.7 deg) was shorter than that between the middle and lower flashes (15.1 deg). We designated this array ‘upper short’. These arrays of flashes simultaneously blinked three times with a duration of 50 ms and ISI of 250 ms, and were followed by a blank display with the duration of 100 ms. Three presentations of flash arrays and the blank display comprised one cycle of stimulus presentation. The duration of one cycle was 1000 ms. During the both pre-test and test phases, infants were presented with 10 cycles on each trial.

#### Procedure

Each infant sat on her or his caregiver's lap in front of a monitor at a viewing distance of approximately 40 cm. Prior to the experiment, caregivers were instructed to close their eyes during the experiment, and were thus naive to the stimuli. Each trial began with the presentation of a colorful fixation figure (approximately 10×10 deg) at the center of the display accompanied by short beeps, which attracted infants' gaze/attention towards the center of the display. A trial started after the fixation figure disappeared and then the experimenter confirmed that the infant was looking at the center of the display. Each infant performed eight trials.

The first and second trials comprised the pre-test phase, in which infants' spontaneous preference for the test stimulus before familiarization was measured (Fig. 1a). The spatial intervals between flashes in each of the two visual arrays were swapped across the two trials. Specifically, when the upper-long and upper-short arrays appeared on the right and left sides in the first trial, the upper-short and upper-long arrays appeared on the right and left sides in the second trial, or vice versa. The order of the trials in the pre-test phase was counterbalanced across infants.

The third-sixth trials comprised the familiarization phase where four stimulus conditions were tested: two directions of flash presentation (upward or downward presentation) and two temporal separations of onset between the second flash and the second beep (+/−150ms lead of beep from flash). We assigned these four conditions to the following categories: upward/−, upward/+, downward/−, and downward/+. Infants were divided into four groups and assigned to an experiment in one of the four conditions.

The seventh and eighth trials comprised the test phase. Procedure was identical to that in the pre-test phase.

It took 10 seconds for each participant to complete a single trial.

#### Data coding and analysis

One observer who was naive to the identity of stimuli in the experiment measured the infants' looking time for each of the right and left flash arrays in each trial in an off-line analysis of video-recordings of the infants' gaze. Specifically, the observer recorded the infants' looking time for the right and left side of the presentation fields by pressing one of two keys while the infant was looking at the relevant field. When the infant looked away from the presentation fields, the looking time was not recorded.

For the familiarization phase, we measured and analyzed infants' total looking time (i.e. the sum of the looking time for right and left presentation fields) in each trial. To test whether the infants habituated to stimuli in the familiarization phase, we compared the sum of looking times for the first and second trials with that for the third and fourth trials using a two-tailed paired t-test.

For the test phase, we compared the proportion of looking time for the novel array to the total looking time for both the novel and familiar arrays. The array with the spatial intervals that were expected to be inconsistent with the perceived spatial interval in the familiarization phase was defined as the *novel visual array*. For instance, if the stimuli in the familiarization phase were expected to cause spatial distortions corresponding to the upper-long array (that is, either in “upward/−” or “downward/+”), the upper-short array was defined as the novel array in the test phase and the upper-long was defined as the familiar array. A two-tailed Student's *t*-test was performed to examine whether the mean proportion of infants' looking time for the novel array was significantly different to that expected by chance (i.e. 0.5). For the pre-test phase, we conducted the same analysis as that used for the test phase in order to confirm whether the preference for novelty in the test phase was due to infants' spontaneous preference for the novel array. Hereafter, we refer to the novel array in the pre-test phase as the ‘virtually novel array’ since the novelty of arrays was later defined on the basis of spatial intervals in the familiarization phase.

### Experiment 2

#### Participants

A total of 24 5- to 8-month-old infants (mean = 213.7 days, standard deviation [SD] = 34.2) took part in Experiment 2. The infants were selected from the participants-database of the infant laboratory, Chuo University (Tokyo, Japan). Local newspaper advertisements were used to recruit infants and their families. Only infants of families who voluntary contacted the infant laboratory were enrolled to the database. All infants were healthy full-terms that weighed >2500 g at birth. Two additional infants took part in this experiment, but their data were excluded from the analysis because experimental sessions were discontinued due to crying and extreme side bias (100% looking to the right side of display) in the test phase.

#### Stimuli

Stimuli in this experiment were identical to those used in Experiment 1 except that the first and third beeps were removed.

#### Procedure, data coding, and analysis

The procedures and analysis were identical to those in Experiment 1.

## Results

### Experiment 1

#### Familiarization phase

**Figure 2:**
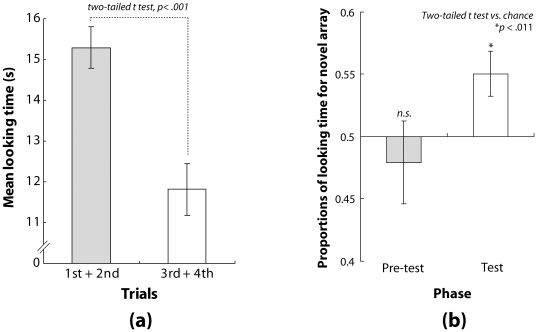
Results of Experiment 1. (a) Mean durations of looking time for the first and second halves of trials in the familiarization phase. Error bars denote standard errors of the means (SEM, N = 24). (b) The left and right bars represent mean proportions of looking time for the novel array in the pre-test and test phases, respectively. Error bars denote SEM (N = 24).

Fig. 2a shows the mean total time for looking at the stimuli in the familiarization phase for the first and second halves of the familiarization phase, respectively. We compared mean total looking times between them in order to assess whether the infants were habituated to the stimuli during the familiarization phase. Mean looking times for the second half of trials would be shorter than those for the first half of trials if infants were habituated to the stimuli in the familiarization phase. The results of a two-tailed t-test revealed that the infants' looking time declined significantly during the familiarization phase (*t*(23) = 6.367, *p*<.001). This indicates that the infants were well habituated to the stimuli in the familiarization phase.

#### Pre-test phase and test phase

Fig. 2b shows the results in the pre-test and test phases. The left bar represents the mean proportion of looking time for the virtually novel array in the pre-test phase. Because there was no reason for the infants to look at either of asymmetrical arrangement of flashes, asymmetrical arrangement of flashes in the test phases would have been equally novel. Hence, in the null hypothesis, the mean proportion looking at either would be deviated from 0.5 (i.e. chance level) due to the unknown factor causing a preference to either of asymmetric arrangement of flashes. In contrast, in the alternative hypothesis, the mean proportion looking at either would be expected around 0.5. As a result, the mean proportion of looking time for the virtually novel array were not significantly different from that expected from chance probability (*t*(23) = −0.624, *p*>.1). The results verified that the infants in Experiment 1 showed no spontaneous preference for either of the spatial patterns in the two flash arrays. The right bar shows the mean proportion of looking time for the novel array in the test phase. If the infants did not experience the audiovisual tau effect in the familiarization phase, asymmetrical arrangement of flashes in the test phases would have been equally novel. Hence, in the null hypothesis, proportion looking at either would be expected around 0.5. In contrast, in the alternative hypothesis, proportion looking at either would be deviated from 0.5 due to the habituation to the audiovisual tau effect. Consequently, the mean proportions of looking at the novel array were significantly higher than would be expected from chance probability (*t*(23) = 2.766, *p*<.011). Taken together with the lack of a significant difference in the proportions of looking time for the virtually novel array in the pre-test phase, the significant difference in the proportions of looking time for the novel array in the test phase suggest that the infants had a preference for the novel array. We conducted an additional analysis using a two-tailed paired t-test to compare the mean proportions of looking time for the virtually novel array in the pre-test phase and the novel array in the test phase. The t-test revealed that the difference in proportions between phases was significant (*t*(23) = 2.369, *p*<.027).

Overall, these results indicate that infants' preferences for the novel array were caused after habituation to the stimuli in the familiarization phase. These results also indicate that perceived spatial intervals between the pairs of flashes in the familiarization phase were distorted for the infants, resulting in a preference for the novel array in the test phase. Moreover, the finding that the direction of spatial distortion was consistent with that in our earlier study of the audiovisual tau effect in adults [19] also indicates that infants, like adults, experience the audiovisual tau effect.

### Experiment 2

The results of Experiment 1 suggest that infants aged between 5–8 months can perceive spatial distortions consistent with the audiovisual tau effect. However, an alternative interpretation for the present results must be considered. Our previous experiment demonstrated that the audiovisual tau effect occurred because temporal intervals of beeps modulated those of flashes, resulting in the distortion of the spatial intervals between flashes by the modulated temporal interval between them [19]. In other words, these previous findings suggested that simple temporal capture of the second flash by the second beep was insufficient to cause the audiovisual tau effect. In the second experiment of this earlier study, an audiovisual tau effect was not observed when the first and third beeps were withdrawn from the stimuli. In order to confirm whether the effect observed in the present Experiment 1 was similar to that observed in the previous study [19], it is necessary to show that removing the first and third beeps from the stimuli reduces the audiovisual tau effect in infancy. Therefore, in this experiment, we used stimuli that were identical to those used in Experiment 1, except that the first and third beeps were removed. Consistent with the previous study [19], any preference for the novel array would be expected to disappear in stimuli without the first and third beeps. On the other hand, it would be expected to be present if perceived spatial intervals between flashes were distorted due to the simple temporal capture of the second pair of flashes by the second beep.

#### Familiarization phase

**Figure 3:**
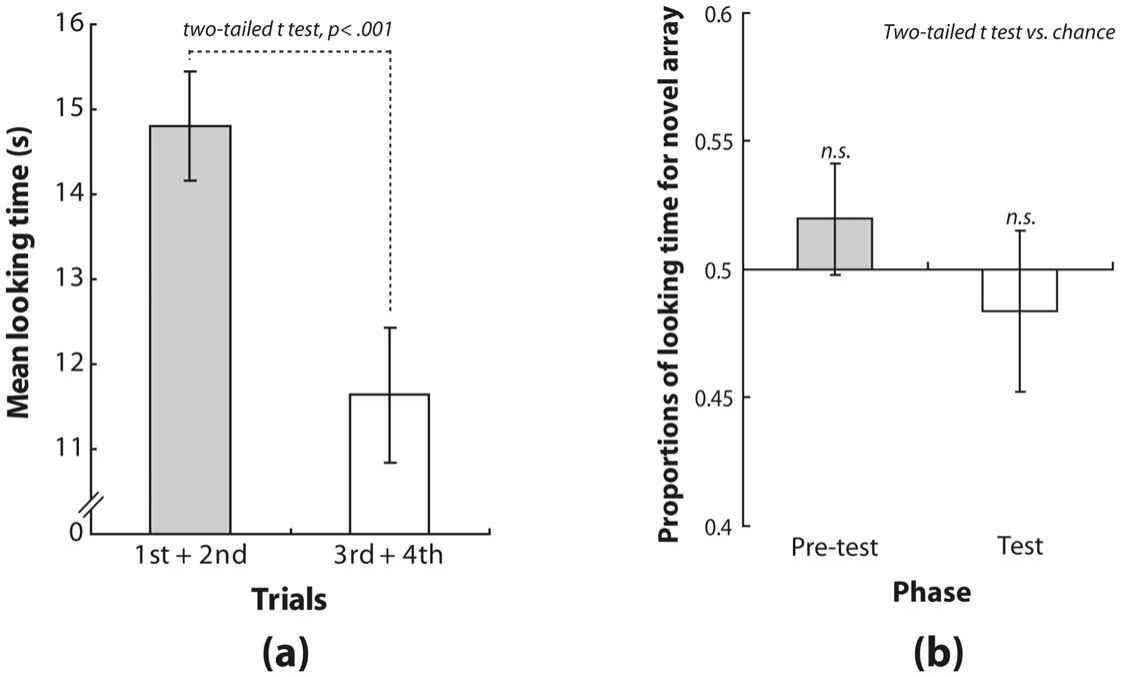
Results of Experiment 2. (a) Mean durations of looking time for the first and second halves of trials in the familiarization phase. Error bars denote SEM (N = 24). (b) The left and right bars represent mean proportions of time spent looking at the novel array in the pre-test and test phases, respectively. Error bars denote SEM (N = 24).

Fig. 3a shows the means of the total looking times for the former two trials and those for the latter two trials. The results indicate that the infants' looking time declined significantly between the first and second half of the trials (*t*(23) = 6.113, *p*<.001). This suggests that the infants were well habituated to the stimuli in the familiarization phase.

#### Pre-test phase and test phase

Fig. 3b shows the results of the pre-test and test phases. The left bar represents the mean proportions of looking time for the virtually novel array in the pre-test phase, which were not significantly different from those expected from chance probability (*t*(23) = −0.913, *p*>.1). The right bar shows the mean proportions of looking time for the novel array in the test phase, which were also not significantly different from chance probability (*t*(23) = −0.515, *p*>.1). A direct comparison of mean proportions of looking times between the pre-test and test phases was performed with a two-tailed paired t-test. This difference was not significant (*t*(23) = −0.908, *p*>.1). These results suggest that the perceived spatial intervals between flashes in this experiment were not distorted. This finding supports the notion that the distortions of spatial intervals between flashes observed in Experiment 1 reflected the audiovisual tau effect, caused by interactions between auditory and visual temporal intervals.

## Discussion

The present study investigated the audiovisual tau effect in infants. In Experiment 1, infants' preferences for the novel array increased significantly following habituation to stimuli in the familiarization phase. In Experiment 2, the infants did not show a preference for the novel array in a condition where the first and third beeps were removed from the stimuli. These results indicate that the preference for the novel array observed in Experiment 1 was due to the audiovisual tau effect, generated by the interaction between the processing for temporal intervals of beeps and flashes. These results are entirely consistent with the features of the audiovisual tau effect reported in our previous study [19]. Taken together, these results suggest that the audiovisual tau effect in both adults and infants may be based on the modulation of the temporal intervals between flashes and beeps, leading to perceptual distortion of the spatial intervals between flashes by modulating the temporal interval between them.

It is noteworthy that this is the first study showing that auditory temporal intervals distort visual temporal intervals even in infants. In adult study, it has been demonstrated that an auditory temporal interval strongly modulated a visual one [19], [28]. However, no previous studies have focused on developmental aspects of audiovisual modulation of temporal interval. Our results demonstrated that the modulation of visual by auditory temporal intervals was established even in infants around 5–8 months of age. However, it is still unclear whether the modulation occurred in infants below 4 months of age, and this was left as an important issue to be challenged in future studies.

The present results also demonstrated that arbitrary interactions between audition and vision were possible in infants around 5–8 months of age. Previous studies have shown that arbitrary interactions among different modalities emerge during the second half of the first year of life [12], [16], [17]. The present data were taken partly from 5 months old infants. Thus, there is a possibility that the audiovisual tau effect, which is one example of arbitrary interactions, occurs during the first half of the first year of life.

Although it was revealed that the audiovisual tau effect occurred even in infancy, critical mechanisms for the audiovisual tau effects in infancy are unclear. Specifically, it is unclear whether the audiovisual tau effect in infancy stems from the interaction between “differentiated” or “undifferentiated” space and time representations. Undifferentiated of space and time representations in the infants predict that a long (or short) temporal interval between flashes is directly represented as a long (or short) spatial interval between flashes. In the present study, it was highly likely that temporal intervals between beeps modulated those between flashes. Upon the undifferentiation, the modulated temporal interval of flashes was directly represented as the modulation of spatial interval of them, resulting in the tau effect. This appears to be a suitable explanation for our observation of the audiovisual tau effect in infancy. However, a recent study with adults suggested that the tau effect is well explained by the Bayesian inference of the spatiotemporal location of stimuli on the basis of differentiated space and time representations [29]. In other words, in adults the spatial location of a flash is estimated on the basis of the interaction between spatial and temporal processing of the flash. Meanwhile, it has also proposed that the representation of time, space and quantity may share common cortical metrics in adults [30]. Thus, at this stage it is difficult to disentangle whether the audiovisual tau effect in infancy (and perhaps in adults) is based on an undifferentiated or differentiated representation of space and time.

The present findings must also be considered in terms of potential mechanisms of multimodal perception in infants. A recent review of the relationship between synesthesia and development of multisensory perception argued that young infants have direct neural connections among different sensory cortexes, and that the incomplete pruning of synapses connecting several sensory cortexes may comprise the neural substrate of synesthesia in adults. It is speculated that the direct connections between visual and auditory cortices underlie the audiovisual tau effect in infants [31]. On the other hand, direct connections, such as those that might underlie the illusory flash effect [32], [33] and flash fusion [34], [35], have been observed in functional magnetic resonance imaging (fMRI) studies of adults [36], [37]. Thus, common neural substrates are likely to underlie the audiovisual tau effect in adults and infants. It will be useful for future work to further examine this possibility.
